# Carbonized Lysine-Nanogels Protect against Infectious Bronchitis Virus

**DOI:** 10.3390/ijms22115415

**Published:** 2021-05-21

**Authors:** Ding-Li Chou, Ju-Yi Mao, Anisha Anand, Han-Jia Lin, John Han-You Lin, Ching-Ping Tseng, Chih-Ching Huang, Hsian-Yu Wang

**Affiliations:** 1 Department of Biological Science and Technology, National Yang Ming Chiao Tung University, Hsinchu 30010, Taiwan; Gs12099@yahoo.com.tw (D.-L.C.); cpts@cc.nctu.edu.tw (C.-P.T.); 2 Department of Bioscience and Biotechnology, National Taiwan Ocean University, Keelung 20224, Taiwan; mao87686@gmail.com (J.-Y.M.); anishaanand84@gmail.com (A.A.); huanging@ntou.edu.tw (C.-C.H.); 3 Center of Excellence for the Oceans, National Taiwan Ocean University, Keelung 20224, Taiwan; 4 School of Veterinary Medicine, National Taiwan University, Taipei 10617, Taiwan; linhanyou@ntu.edu.tw; 5 School of Pharmacy, College of Pharmacy, Kaohsiung Medical University, Kaohsiung 80708, Taiwan; 6 Graduate Institute of Animal Vaccine Technology, College of Veterinary Medicine, National Pingtung University of Science and Technology, Pingtung 91201, Taiwan

**Keywords:** lysine, carbonization, nanogels, infectious bronchitis virus, antiviral agents

## Abstract

In this study, we demonstrate the synthesis of carbonized nanogels (CNGs) from an amino acid (lysine hydrochloride) using a simple pyrolysis method, resulting in effective viral inhibition properties against infectious bronchitis virus (IBV). The viral inhibition of CNGs was studied using both in vitro (bovine ephemeral fever virus (BEFV) and pseudorabies virus (PRV)) and in ovo (IBV) models, which indicated that the CNGs were able to prevent virus attachment on the cell membrane and penetration into the cell. A very low concentration of 30 μg mL^−1^ was found to be effective (>98% inhibition) in IBV-infected chicken embryos. The hatching rate and pathology of IBV-infected chicken embryos were greatly improved in the presence of CNGs. CNGs with distinctive virus-neutralizing activities show great potential as a virostatic agent to prevent the spread of avian viruses and to alleviate the pathology of infected avian species.

## 1. Introduction

Infectious bronchitis virus (IBV), a chicken-affecting coronavirus, is a major pathogen that causes significant economic losses in the poultry industry due to poor weight gain and downgrading of eggs in egg-laying hens [1]. Though IBV causes mucosal respiratory infection, depending on the viral strain, it can affect the epithelial cells of the digestive, reproductive and urinary tracts, which then spreads to other organs [2]. IBV belongs to the *Gammacoronavirus* genus within the Coronaviridae family, with a non-segmented, positive-sense single-stranded RNA genome and an envelope [1]. Similarly to the other members in Coronaviridae, IBV shows continual mutation of antigenic variants, with numerous serotypes in different parts of the world [3]. Vaccination is the major method of IBV infection control however, it is only partially successful due to antigenic variation [4]. Although the use of the cross-protective attenuated H120 strain with other local isolated virus strains may provide better disease prevention, the natural characteristics of the coronavirus and the cross-infection issues result in occasional outbreaks [5]. Natural extracts such as essential oils, saponin, phenylpropanoid glycoside, and polyphenols have been studied as antiviral agents to prevent or control IBV infection in chickens in recent years [6,7,8,9,10]. However, such phytochemicals are not effective in providing complete protection of chickens from IBV infection.

Nanoparticle-based antiviral agents have been reported for application in antiviral therapeutics in recent years, due to their distinctive intrinsic antiviral activities, targeting, or drug delivery efficacy [11]. Carbon-based nanomaterials, such as surface-functionalized graphene oxides, carbon nanotubes, and carbon dots, have shown their effectiveness against various types of viral infections, including enterovirus, influenza virus, human immunodeficiency virus, and human alphaherpesvirus [12,13,14,15,16,17]. Notably, the highly biocompatible carbon dots, developed using different precursors (e.g., organic salt, amino acids, polymers) or different methods (e.g., hydrothermal, microwave, electrochemistry, or pyrolysis), act as disparate inhibitors at different stages of viral infection, including virus attachment, penetration, replication, and budding [18,19,20,21,22,23]. Most reported carbon dots have been demonstrated to interact with the viral surface protein and inhibit their penetration into host cells [18,19,20]. On the other hand, the inhibition of viral replication by carbon dots is accomplished by the alteration of enzymes that are essential for viral genome replication and finally for budding [12,21,22,23]. However, most studies overlook the role of polymeric structures on the surface of carbon dots in their interaction with the virus; thus, we were motivated to reveal the flexible polymeric carbon nanomaterials underpinning the action of these dots in the treatment of viral infections.

Polylysine, the homo-polymer of lysine with a positively charged synthetic amino acid chain, is widely used as a coating to enhance cell attachment and adhesion [24]. Polylysine has also been widely used as an antibacterial agent due to its broad-spectrum antimicrobial activity and low toxicity [25]. In addition, polylysine has showen mild antiviral activity, although the mechanism is unclear [26]. In this study, we aimed to synthesize antiviral carbonized nanogels (CNGs) through a simple pyrolysis treatment of lysine hydrochloride. Dry-heating of lysine hydrochloride at different temperatures (180 °C–300 °C) resulted in the formation of CNGs with different degrees of carbonization and surface passivation of polylysine-like structures. The structure and the antiviral potency of the CNGs were highly correlated with their synthesis temperature. The in vitro cytotoxicity and hemolysis assessment of the CNGs revealed high biocompatibility. Compared to natural lysine, the CNGs exhibited outstanding antiviral activity against IBV. The inhibition of IBV by the CNGs is mainly caused by the blocking of the virus attachment to the host cell. We conducted an in ovo study of chicken embryos infected with IBV as the model in avian species, which revealed an improved survival rate and protective effect from morbidity in CNG-treated chicken embryos. Our results suggested that the highly biocompatible CNGs might be a potential candidate for therapeutic use against IBV coronavirus infection.

## 2. Results

### 2.1. Characterization of CNGs

**Figure 1:**
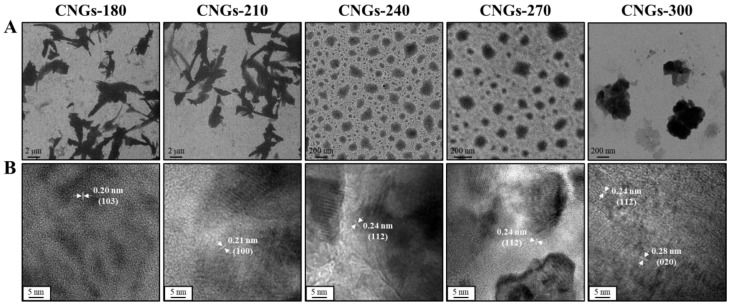
(**A**) TEM and (**B**) HRTEM images of CNGs synthesized at 180 °C–300 °C.

CNGs from lysine hydrochloride were prepared by directly pyrolyzing them at 180 °C, 210 °C, 240 °C, 270 °C, or 300 °C for 2 h in the solid-state. These were purified and were denoted as CNGs-180, CNGs-210, CNGs-240, CNGs-270, and CNGs-300_,_ respectively. The product yields were decreased from 96.5 to 20.5% with the increase in the heating temperature from 180 °C to 300 °C (Table S1). The CNGs exhibited good water dispersibility at room temperature, except for CNGs-300, due to over-carbonization (Figure S1) [27]. The hydrodynamic diameters of CNGs synthesized at 180 °C to 270 °C show a decrease from ca. 510 nm to 120 nm, due to higher condensation and carbonization with increasing temperature. Compared to CNGs-270, the larger hydrodynamic size of CNGs-300 (ca. 280 nm) is due to the higher degree of carbonization, resulting in larger carbon particles and low product yield (Table S1). Transmission electron microscopy (TEM) images of CNGs further confirmed the morphology and size, which were in agreement with the dynamic light scattering (DLS) results (Figure 1A). CNGs were formed as distinct particles, along with self-assembled polypeptide structures and polymer matrices, when lysine was heated at 180 °C–300 °C due to polymerization, condensation, and carbonization. The CNGs-180 and CNGs-210 in the TEM images showed irregular polymer-like particles with a mean size of ca. 510 and 300 nm, respectively, from 50 counts, then the size decreased with the increasing temperature. The polydispersity index (PDI) values of the CNGs ranged from 0.4 to 0.7 (Table S1), indicating that CNGs are polydisperse, with a broad distribution. Notably, CNGs-240 and CNGs-270 appeared as well-distributed nanogel-like carbonized particles with a size of ca. 135 and 100 nm, respectively. In contrast, CNGs-300 appeared as large-sized carbon residues on the TEM image (ca. 220 nm). The HRTEM images of all the CNGs showed varying *d*-spacing values of 0.20, 0.21, 0.24, and 0.28 nm, attributed to the lattice planes of the (103), (100), (112), and (020) facets of the graphitic structures, respectively, revealing the formation of crystalline graphene-like structures (Figure 1B) [28]. Furthermore, prominent crystal structures were developed in the CNGs formed at higher temperatures.

The CNGs exhibited ζ-potential values from ca. +10 to +25 mV (Table S1), as a result of the preservation of high amine content during dehydration, condensation, and carbonization of the carboxylic acid group, α-amino group, amino group on the side chain, and the alkyl group. The pyrolyzed lysine probably forms pyridine, lactams, piperidines, pyrroles, amides, tertiary amine, hexamethyleneimine, and cyclohexanone residues, which are incorporated into the CNGs [29]. The UV-visible absorption spectra of CNGs showed a strong absorption band at 270–290 nm due to the π→π* transitions of conjugated C=C bonds (Figure S2) [30]. A shoulder band at 310–350 nm is ascribed to the n→π* surface and edge defect transitions of CNGs with C=O bonds, C=N bonds, or the interlayer π→π* charge transfer, revealing oxygen- and nitrogen-containing functional groups in the CNGs [30]. Upon excitation at 365 nm, the CNGs emitted a strong and broad fluorescence band centered at a wavelength of ca. 450 nm. Nitrogen-doped CNGs usually exhibit strong fluorescence, which could be attributed to the formation of diverse sizes of polycyclic aromatic clusters, edge defects, surface emissive traps, and restriction of the intramolecular vibrations and rotations, thereby minimizing the internal non-radiative relaxation pathways [31]. The fluorescence quantum yields (QYs) of the CNGs are presented in Table S1, which shows that CNGs-300 had the highest QY (17.3%) of all, probably due to it having the highest degree of carbonization. The elemental composition (C, H, O, N, and Cl) of the CNGs, presented in Table S2, shows that the carbon content (40.1–60.3%) of the CNGs increased with increasing temperature, indicating a carbonization process at high temperatures. In contrast, the oxygen (9.3–23.7%) content of the CNGs showed an opposite trend with increasing temperature, due to the oxygen loss during the dry-heating process. Notably, CNGs-270 showed the highest nitrogen content (16.6%), implying the formation of more pyridine-like structures at 270 °C [32]. The X-ray diffraction (XRD) patterns of lysine, purified CNGs-180, and CNGs-210 showed some sharp peaks and a broad-band at ca. 18–33°, which could be ascribed to the characteristic peaks of molecular lysine crystals (Figure S3) [33]. The characteristic diffraction peak gradually decreased and disappeared with the increment in synthesis temperature, due to partial condensation and carbonization. The XRD patterns of purified CNGs-240, CNGs-270, and CNGs-300 showed a broad band centered at ca. 26° (2θ), which could be ascribed to the interlayer spacing (002) in bulk graphite (0.33 nm) [34]. The FT-IR spectra of purified CNGs-180 and CNGs-210 revealed the presence of discernible characteristics of lysine (Figure S4). When the synthesis temperature increased from 240 °C to 300 °C, C–H stretching (2853 cm^–1^), C=C=N stretching (2000 cm^–1^), C=O stretching (1760 cm^–1^), C=C stretching (1662 cm^–1^), and pyridinic-N–oxide stretching (1520 cm^–1^) increased significantly, indicating significant carbonization and the formation of new functional groups.

### 2.2. Antiviral Activity of CNGs

**Figure 2:**
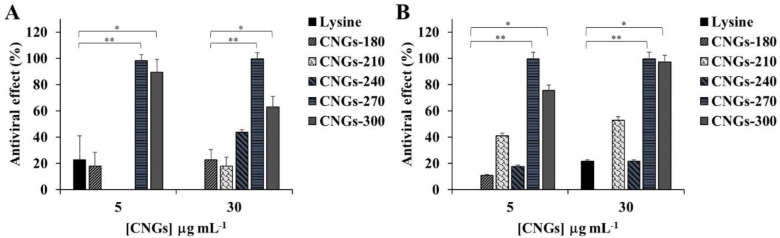
Inhibitory effects of lysine hydrochloride or CNGs on (**A**) BEFV-infected BHK-21 cells and (**B**) PRV-infected Vero cells. The viruses and CNG mixtures were inoculated into BHK-21 or Vero cells for infection. After 30 min at 37 °C incubation, the inoculum was replaced with growth media and incubated for 72 h before the calculation of antiviral effects. The error bars represent standard deviations for three independent experiments. The results were analyzed using ANOVA multiple comparisons (* *p* < 0.05, ** *p* < 0.01).

To evaluate the virus-infection inhibiting effects of the CNGs, two enveloped viruses, bovine ephemeral fever virus (BEFV) and pseudorabies virus (PRV), were incubated with CNGs and then infected with BHK-21 and Vero cells, respectively. The viruses (10^6.5^ TCID_50_ mL^–1^) were incubated with 5 or 30 μg mL^–1^ of CNGs for 30 min at room temperature before dilution and were inoculated into the cultured cells. MEM media without CNGs served as the virus-positive control group. The mixtures of the virus and CNGs were removed after 30 min of the virus inoculation process. The antiviral effects of CNGs were calculated as described in the experimental section, and the data showed that BEFV was only significantly inhibited by CNGs-270 and CNGs-300 at both 5 and 30 μg mL^–1^ concentrations (Figure 2A). The other CNGs showed mild antiviral potency. Similar results were observed for the inhibition of PRV in Vero cells (Figure 2B). On the other hand, the CNGs showed low cytotoxic effects (viability>70%) on both cell lines, even with concentrations up to 50 μg mL^–1^ (Figure S5). CNGs-300 showed significant inhibition of BEFV and PRV in vitro in a concentration-independent manner, mainly because the CNGs caused slight cytotoxicity to BHK-21 cells and Vero cells at a high concentration of 30 µg mL^–1^. CNGs-300 are highly carbonized nanoparticles with well-defined graphitic structures with nitrogen doping (Figure 1B, Figures S3 and S4). Nitrogen-doped carbon nanoparticles are reported to exhibit dose-dependent moderate cytotoxicity by inducing the generation of reactive oxygen species [35]. In addition, incubation of CNGs with the cells in media for 24 h at 37 °C indicated no significant cytotoxicity for all CNGs up to 50 µg mL^–1^, except for CNGs-300 (Figure S6). However, all the CNGs showed significant cytotoxicity when incubated for 72 h, mainly due to the low concentration of the FBS (2%) used in the cell culture. In addition, no significantly hemolytic effect was observed for CNGs-270 even up to 100 μg mL^–1^ (Figure S7). Furthermore, the microscopic images of the CNG-treated BHK-21 and Vero cells show typical morphological characteristics, without a noticeable unhealthy cell population (Figure S8).

The HRTEM images of the BEFV and PRV viruses treated with CNGs-270 suggest significant adsorption of CNGs on the virus envelope (Figure S9), identified based on the lattice fringes of the CNGs in the images. Therefore, the adsorption of the CNGs may have hindered the virus attachment to the mammalian cells. Our previous study on the inhibition of enterovirus by carbon dots obtained from curcumin revealed that carbonized nanoparticles have a high affinity toward the structural proteins of the virus [18]. In this study, the slightly positively charged CNGs may bind to the spike glycoprotein of the viruses to exert their antiviral effects. The CNGs-270 and CNGs-300, with a higher degree of carbonization, showed stronger antiviral effects than other CNGs, probably due to their strong interaction with proteins (e.g., glycoprotein and spike protein) through electrostatic force, π–π stacking interaction, Van der Waals forces, and hydrogen bonding [36,37,38].

**Figure 3:**
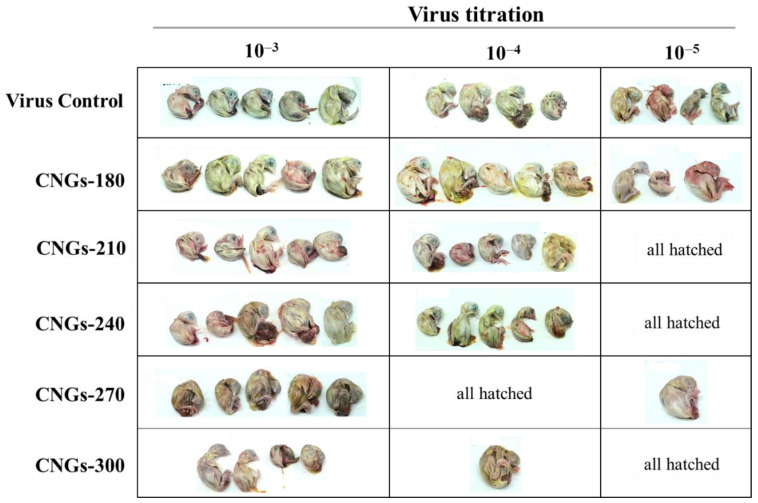
Image records of IBV-infected chicken embryos in the absence or presence of CNGs. IBV (10^6.25^ CEID_50_ mL^–1^) was pre-incubated with CNGs (30 μg mL^–1^) for 30 min at room temperature and inoculated into 13-day-old chicken embryos (*n* = 5). The pathological effect was recorded after 7 days of incubation. The chickens in negative controls were all hatched after 7 days of incubation and are not shown here.

**Figure 4:**
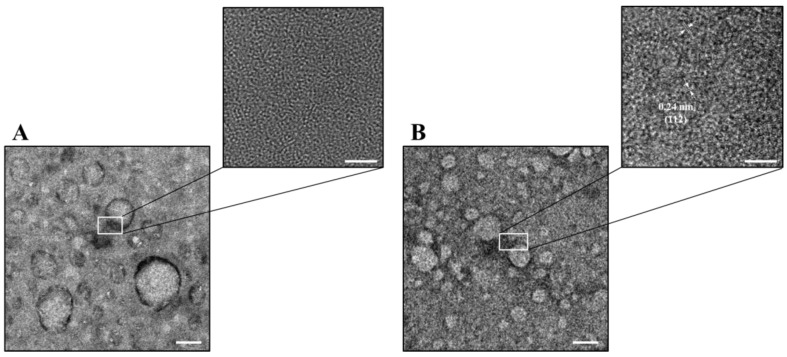
TEM and HRTEM images of IBV (5 × 10^5.25^ CEID_50_ mL^–1^) (**A**) before and (**B**) after incubation with CNGs-270 (1.0 μg mL^–1^) for 30 min in PBS. Scale bars in the TEM and HRTEM images are 100 and 5 nm, respectively.

To further study the antiviral effects of CNGs, chicken embryos infected with IBV were used as the in ovo model. The serially diluted IBV (10^6.25^ CEID_50_ mL^–1^) was incubated with 30 μg mL^–1^ CNGs and inoculated into a 13-day-old chicken embryo. After 7 days of incubation, the hatching rate and chicken embryo pathological effects were recorded for the evaluation of the antiviral activity of CNGs in ovo. Although PRV, BEFV, and IBV belong to the enveloped virus, the virus-induced pathology could be better understood in IBV-infected chicken *embryos* in the in ovo model. PRV and BEFV are not able to infect chicken embryos. The IBV-infected chicken embryo (control group; IBV without preincubation with CNGs) showed hemorrhaging on the legs, liver necrosis, kidney swelling, dwarfing, and curling (Figure 3). CNGs-180 showed a negligible protective effect, similar to that of the control group. The antiviral effects of CNGs-210, CNGs-240, CNGs-270, and CNGs-300 were determined to be 82.2%, 82.2%, 97.8%, and 98.2%, respectively, determined based on the relative 50% chicken embryo infectious dose (CEID_50_) (Table 1). The CNGs were able to significantly suppress the IBV infection rate in ovo. The TEM images displayed in Figure 4 demonstrate that the antiviral effect of CNGs occurred mainly because of the binding of CNGs onto IBV particles, leading to the inhibition of viral attachment to the host cell. The glycoprotein of IBV has two major domains, S1 and S2, which are involved in receptor attachment and membrane fusion, respectively, with the host cells [39]. The polymeric CNGs adsorbed on virus particles may hinder its S1 and S2 proteins to interact with host cells for infection. The CNGs synthesized at higher temperatures possess a higher positive charge, which may neutralize the surface charge of IBV and attenuate the pathogenicity of IBV. Although CNGs-270 and CNGs-300 showed good inhibition of PRV and BEFV infections, CNGs-270 showed the best antiviral activity in the IBV-infected in ovo model, probably due to the appropriate particle size or functional groups. On the other hand, all CNGs did not exhibit acute toxicity toward the chicken embryo; the eggs were all hatched in the groups with low concentrations of CNGs. Therefore, the adverse effect on the development of chicken embryos at higher concentrations of viruses is mainly due to the virus, rather than CNGs.

## 3. Discussion

In summary, we have synthesized CNGs with distinguishing antiviral properties, from lysine hydrochloride, using a simple pyrolysis method. The CNGs exhibited excellent antiviral properties against BEFV and PRV in vitro and IBV in ovo. The antiviral activity was found to increase with the increment in the synthesis temperature, probably due to the formation of more graphene-like structures at higher temperatures. We hypothesize that the antiviral properties of the CNGs against the three enveloped viruses (e.g., PRV, BEFV, and IBV) are due to the adsorption of CNGs on the spike glycoprotein of the viruses, resulting in the inhibition of receptor attachment and membrane fusion. Therefore, our CNGs show great potential to prevent the spread of enveloped viruses, such as coronavirus. Although some CNGs show slight toxicity to test cells, they did not affect the hatching rates of chicken eggs. Therefore, we suggest that a moderate incubation period and concentration of the CNGs is necessary. We will investigate the accumulation of CNGs in tissues and organs, and their long-term adverse effects, in our future work.

## 4. Materials and Methods

### 4.1. Synthesis and Characterization of CNGs

The CNGs were synthesized from lysine hydrochloride using a simple pyrolysis method. Briefly, 500 mg of lysine hydrochloride was placed in a 50 mL glass vial and dry heated in a laboratory-grade convection oven (DH 300, Dengyng, Taiwan) at constant temperatures of 180 °C, 210 °C, 240 °C, 270 °C, or 300 °C for 2 h and the CNGs were labeled as CNGs-180, CNGs-210, CNGs-240, CNGs-270, CNGs-300, respectively. The solid product obtained was allowed to cool to room temperature and then dispersed in 40.0 mL of deionized water by sonication (DC 200, DELTA, Taiwan) for 3 h. The larger particles were removed by centrifugation at a relative centrifugal force (RCF) of 35,000× *g* for 1 h. The as-obtained CNG dispersions were stored at 4 °C for future use. A CNG dispersion in a physiological buffer solution was used for the antiviral assay.

The hydrodynamic diameters and zeta potential (ζ-potential) of CNGs in 5 mM sodium phosphate buffer (pH 7.4) were measured by means of dynamic light scattering (DLS) using a Zetasizer 3000HS analyzer (Malvern Instruments, Malvern, UK). The particle size and morphology of the CNGs were analyzed using Tecnai G2 F20 S-TWIN (Philips/FEI, Hillsboro, OG, USA) transmission electron microscopy (TEM) systems, operating at 200 kV. The CNGs were carefully deposited on 300-mesh formvar/carbon-coated Cu grids at ambient temperature. The fluorescence spectra of the CNG dispersions in 5 mM sodium phosphate buffer (pH 7.4) were recorded using a monochromatic microplate spectrophotometer (Synergy^TM^ 4 Multi-Mode; BioTek Instruments, Winooski, VT, USA). The fluorescence quantum yields (QYs) of the CNGs were determined by comparison with that of quinine sulfate standard (QY = 54% in 0.1 M H_2_SO_4_). Elemental analysis (EA) was performed using a Vario EL cube analyzer (Elementar, Hanau, Germany) for C, H, O, and N. Samples for X-ray diffraction (XRD) were prepared by depositing the CNG solution on the silicon wafer and drying at 50 °C for 12 h. XRD measurements were carried out at room temperature using an X-ray diffractometer (D/MAX 2200 VPC, Rigaku, Sendagaya, ShibuyaKu, Tokyo, Japan) with the Cu *K*_α1_ line (λ = 1.54 Å, energy = 8.8 keV). A Fourier transform infrared spectrometer (FT-IR, FT/IR-6100, JASCO, USA) in transmission mode in the range of 500 to 4000 cm^–1^ with 32 scans was used to identify the possible functional groups existing in the CNGs. High-purity nitrogen was used for purging during the FT-IR measurements to minimize the interference of water vapor.

### 4.2. Cytotoxicity Assays

The BHK-21 (fibroblast cells from baby hamster kidneys) and Vero (monkey kidney epithelial cells) cells were pre-cultured in 96-well culture plates (1 × 10^4^ cells well^–1^) for 24 h. Lysine hydrochloride (without heating) or CNGs were diluted to the required concentration (30–50 μg mL^–1^) in media, added to the cells, and incubated for 30 min. The CNG-containing media were replaced by the fresh media with 2% FBS. After 72-h culture at 37 °C, the cell survival rate was analyzed by means of neutral red live-cell staining and OD_540_ was determined. We tested the cytotoxicity of the CNGs by incubating them with the cell lines in media for 24 h.

### 4.3. Antiviral Assays

The BHK-21 cells and Vero cells were seeded in the 96-well plate (1 × 10^4^ cells well^–1^) and cultured overnight. The BEFV and PRV virus seeds (10^6.5^ TCID_50_ mL^–1^; 50% Tissue Culture Infectious Dose) were preincubated with the CNG samples (5 or 30 μg mL^–1^) in MEM at room temperature for 30 min. The virus-positive control was incubated with the MEM at the same time. The CNGs and virus mixtures were serially diluted and inoculated into BHK-21 or Vero cells, respectively. After 30 min of incubation at 37 °C, the inoculum solution was replaced by the growth media with 2% FBS. The TCID_50_ results were determined using the Reed–Muench method after 72 h of culture. The antiviral effects of CNGs were calculated as per the formula:

Antiviral effect (%) = [(TCID_50_ of control group) − (TCID_50_ of CNGs group)]/(TCID_50_ of control group) ×100%.

### 4.4. Hemolysis Assay

Blood samples for the hemolysis assay were collected from female 2-month-old Sprague-Dawley rats in tubes containing ethylenediaminetetraacetic acid (EDTA) and immediately (within 30 min of the collection) centrifuged (RCF 3000× *g*, 10 min, 4 °C) to remove the serum and then diluted with sterile isotonic physiological buffer (ca. 4.0 vol% blood cells). For the analysis, 200 µL of the RBC stock suspension was incubated with aliquots of CNGs-270 dispersions (1–100 μg mL^–1^) in 1.5-mL vials at 37 °C for 1 h, followed by centrifugation at an RCF of 1000 *g* for 10 min. Hemolysis was measured based on the absorption of hemoglobin at 576 nm (OD_576_) in the supernatant (150 µL). Hemolysis in sterile isotonic physiological buffer and ultrapure water was used as negative and positive control, respectively, and the hemolysis activity was calculated as:

Hemolysis (%) = [(OD_576 CNGs-270_ − OD_576 blank_)/(OD_576 ultrapure water_ − OD_576 blank_)] × 100%.

### 4.5. In Ovo Antiviral Test in Chicken Embryo against IBV Infection

The animal experiments were approved by the Institutional Animal Care and Use Committee of National Pingtung University of Science and Technology. The project identification codes are NPUST-108-039 and NPUST-110-044, date of approval: 25 June 2019. The IBV virus (10^6.25^ CEID_50_ mL^–1^) was incubated with the CNG samples (final concentration = 30 μg mL^–1^) in phosphate-buffered saline (PBS; pH 7.4, containing 137 mM NaCl, 2.7 mM KCl, 10 mM Na_2_HPO_4_, and 2.0 mM KH_2_PO_4_) at room temperature for 1 h. The virus-positive control was incubated with the PBS at the same time. The virus-CNG inoculum was injected into the 13-day-old SPF chicken embryos. The embryo pathological effects were determined after 7 days of incubation (37 °C, 40% humidity). The 50% chicken embryo infectious dose (CEID_50_) results were determined using the Reed–Muench method. The antiviral effects of CNGs were calculated as per the formula:

Antiviral effect (%) = [(CEID_50_ of control group) − (CEID_50_ of CNGs group)]/(CEID_50_ of control group) × 100%.

## Figures and Tables

**Table 1:** CEID_50_ mL^–1^ and antiviral effect (%) of CNGs against IBV infection in the chicken embryo.

Group	Control *^a^*	CNGs-180	CNGs-210	CNGs-240	CNGs-270	CNGs-300
**CEID_50_ mL** **^–1^**	10^6.25^	10^6.25^	10^5.50^	10^5.50^	10^4.60^	10^4.50^
**Antiviral effect (%) *^c^***	NA *^b^*	0%	82.2%	82.2%	97.8%	98.2%

*^a^* IBV infection only (IBV without preincubation with CNGs). *^b^* Not assayed. ^*c*^ Antiviral effect (%) = ((CEID_50_ of control group) − (CEID_50_ of CNG group))/(CEID_50_ of control group) × 100%.

## Supplementary Materials

The following are available online at https://www.mdpi.com/article/10.3390/ijms22115415/s1.
