# Supplementary material for: Carbonized Lysine-Nanogels Protect against Infectious Bronchitis Virus
Source: Int J Mol Sci. 2021 May 21;22(11):5415. doi: 10.3390/ijms22115415 (PMC8196573; doi:10.3390/ijms22115415)
Supplement: Supplementary file 1 [file ijms-22-05415-s001.zip › ijms-1208288 supplementary.pdf]

Materials and Methods

Materials. Lysine monohydrochloride, hydrochloric acid (HCl), sodium chloride (NaCl), potassium chloride (KCl), magnesium chloride ($MgCl_2$), calcium chloride ($CaCl_2$), and tris(hydroxymethyl)aminomethane (Tris) were purchased from Sigma-Aldrich (St. Louis, MO, USA). Phosphoric acid and acetic acid were purchased from Mallinckrodt Baker (Phillipsburg, NJ, USA). All the chemicals were used without modification. Phosphate buffered saline (PBS; containing 137 mM NaCl, 2.7 mM KCl, 10 mM Na_2HPO_4, and 1.8 mM KH_2PO_4; pH 7.4) was used to mimic physiological conditions. Milli-Q ultrapure water (18.2 MΩ·cm; EMD Millipore, Billerica, MA, USA) was used in all experiments.

Cells and viruses. BHK-21 cells (BCRC#60041) and Vero cells (BCRC#60013) were purchased from the Bioresource Collection and Research Center, Taiwan. Both cells were cultured with Eagle's Minimum Essential Medium (MEM, Gibco, NY, USA) supplemented with 10% fetal bovine serum (FBS, Gibco, NY, USA), at 37 ℃ in an atmosphere of 5% CO_2. The BEFV (Tn88128 strain), PRV (PRV-TNL strain), and IBV (M41 strain) were provided by Dr. C.-Y. Chu (National Pingtung University of Science and Technology, Taiwan). The BEFV and PRV were cultured in BHK-21 and Vero cells respectively. The IBV was cultured in specific pathogen-free (SPF) chicken embryos (Animal Health Research Institute, COA, Taiwan) at 37 ℃, relative humidity of 40–70%.

Table S1. Product yield, polydispersity index, hydrodynamic diameter, zeta potential, and fluorescence quantum yield (QY) of the as-prepared products from lysine hydrochloride by dry heating.

	**Product yield (%)**[a]	**PDI**[b]	**Hydrodynamic diameter (nm; *n*=5)**	**Zeta potential (mV; *n*=5)**	**Fluorescence QY (%)**[c]
CNGs-180	96.5	0.72	506.2 ± 246.6	10.3 ± 3.4	4.1
CNGs-210	89.5	0.51	313.5 ± 85.6	11.2 ± 3.9	9.8
CNGs-240	80.4	0.45	156.8 ± 43.2	20.1 ± 1.9	16.1
CNGs-270	61.5	0.43	122.6 ± 29.9	24.9 ± 1.4	9.9
CNGs-300	20.5	0.49	278.6 ± 35.5	18.9 ± 3.3	17.3

[a] determined after the purification process by centrifugation of as-prepared products.

[b] polydispersity index (PDI).

[c] compared to quinine sulfate (QY: 54% in 0.1 M H_2SO_4).

Table S2. Elemental compositions of lysine and the CNGs were obtained by dry heating of lysine hydrochloride at 180, 210, 240, 270, and 300 ℃ for 2 h.

	**Elemental compositions (wt%)[a]**				
	**C (%)**	**H (%)**	**O (%)**	**N (%)**	**Cl (%)[b]**
Lysine hydrochloride[c]	38.5	8.4	18.4	15.3	19.5
CNGs-180	40.1	7.2	23.7	13.7	15.3
CNGs-210	40.5	8.9	20.3	14.3	16.0
CNGs-240	42.3	7.4	15.2	16.0	19.1
CNGs-270	45.5	8.2	9.3	16.6	20.4
CNGs-300	60.3	7.4	14.0	11.0	7.3

[a] determined by elemental analysis.

[b] determined by ICP-MS.

[c] theoretical value of lysine hydrochloride ($C_6H_{15}ClN_2O_2$): C (39.5%), H (8.2%), O (17.6%), N (15.4%), and Cl (19.3%).

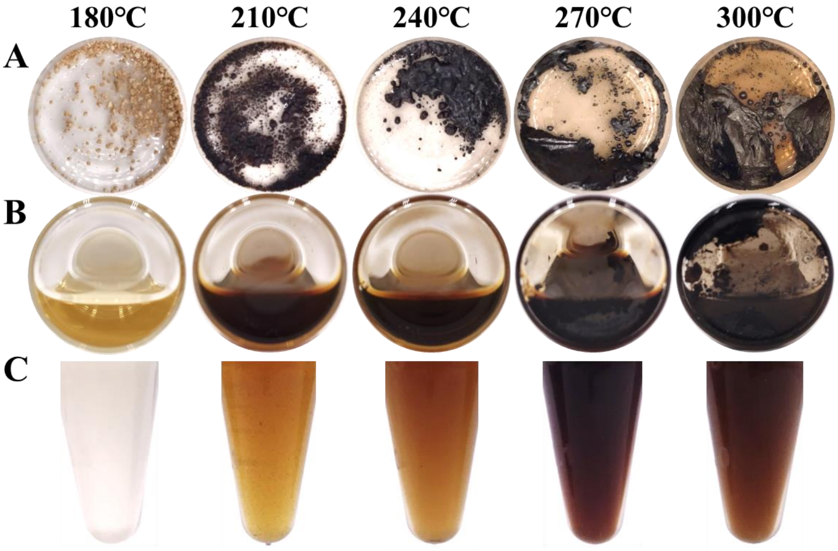

Figure S1. Photographs of the as-obtained CNGs. (A) Dry heating (180–300 ℃) of lysine hydrochloride for 2 h, (B) dispersion of the obtained residue in deionized water by sonication for 3 h, and (C) purified CNGs dispersed in deionized water. The CNGs were purified by centrifugation with a relative centrifugation force (RCF) of 5,000 *g* for 60 min to remove aggregated nanoparticles.

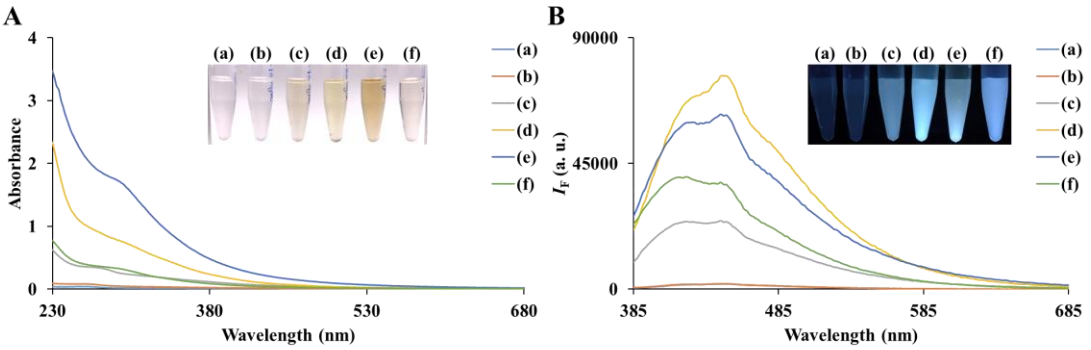

Figure S2. (A) UV-vis absorption and (B) fluorescence spectra of CNGs. (a) lysine hydrochloride without heating, (b) CNGs-180, (c) CNGs-210, (d) CNGs-240, (e) CNGs-270, and (f) CNGs-300. All purified samples were 20-fold diluted in 5 mM sodium phosphate buffer (pH 7.4) for measurements. Insets to (A) and (B) are the photographs of the corresponding solutions after 10-fold dilution under visible light and excitation with a UV light of 365 nm. Fluorescence spectra of all samples were recorded at an excitation wavelength of 365 nm. The fluorescence (I_F) intensities are plotted in arbitrary units (a. u.).

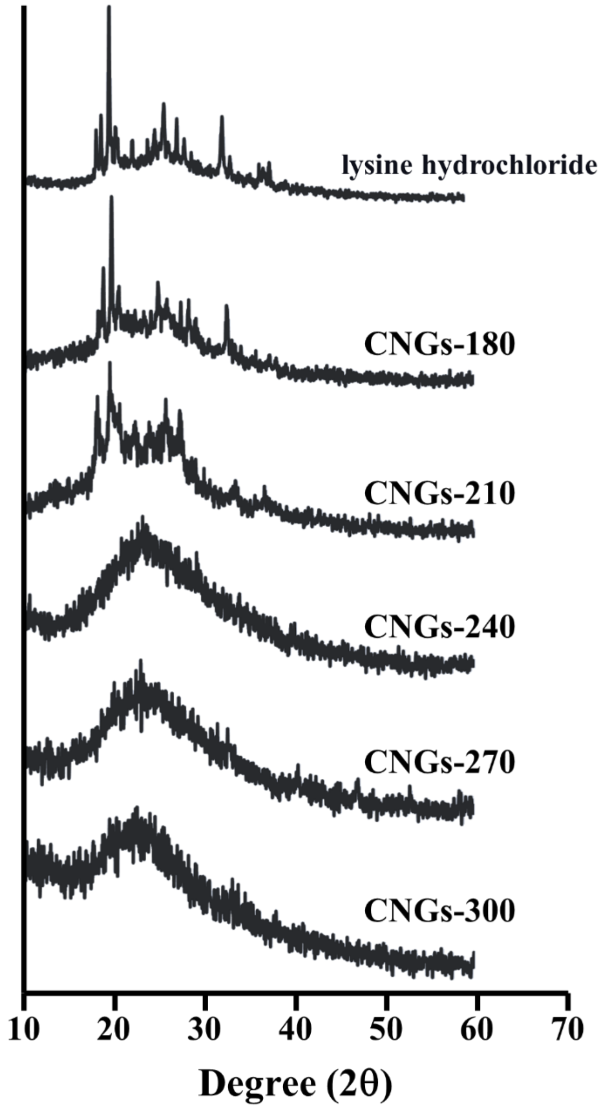

Figures S3. XRD spectra of lysine hydrochloride and CNGs synthesized at 180–300 ℃.

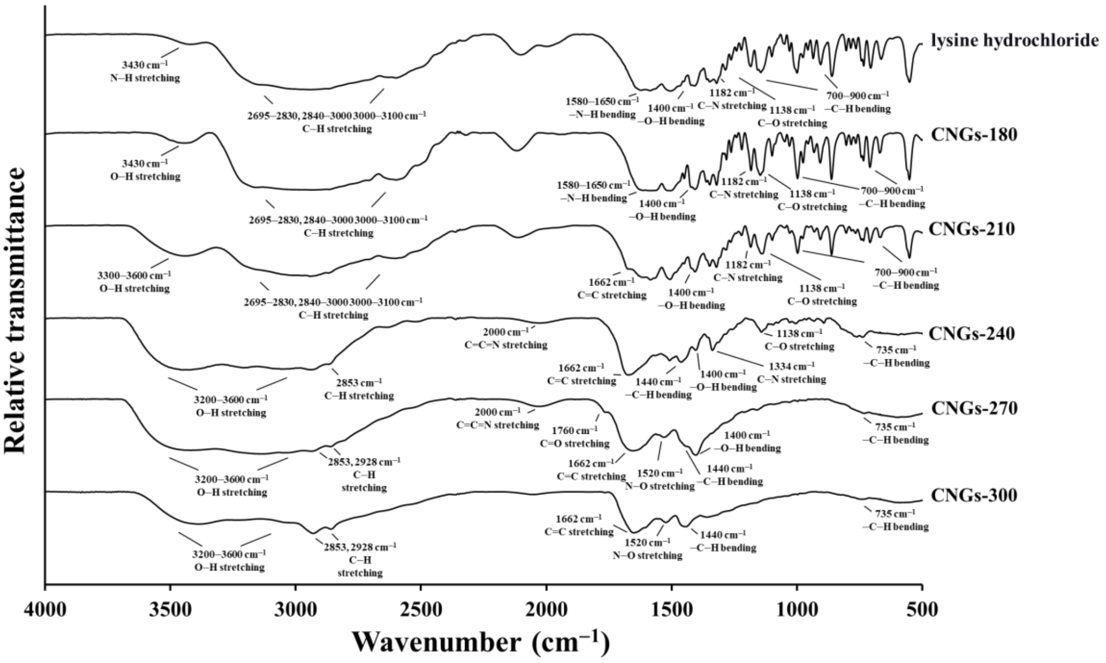

Figures S4. FT-IR spectra of lysine hydrochloride and CNGs synthesized at 180–300 ℃.

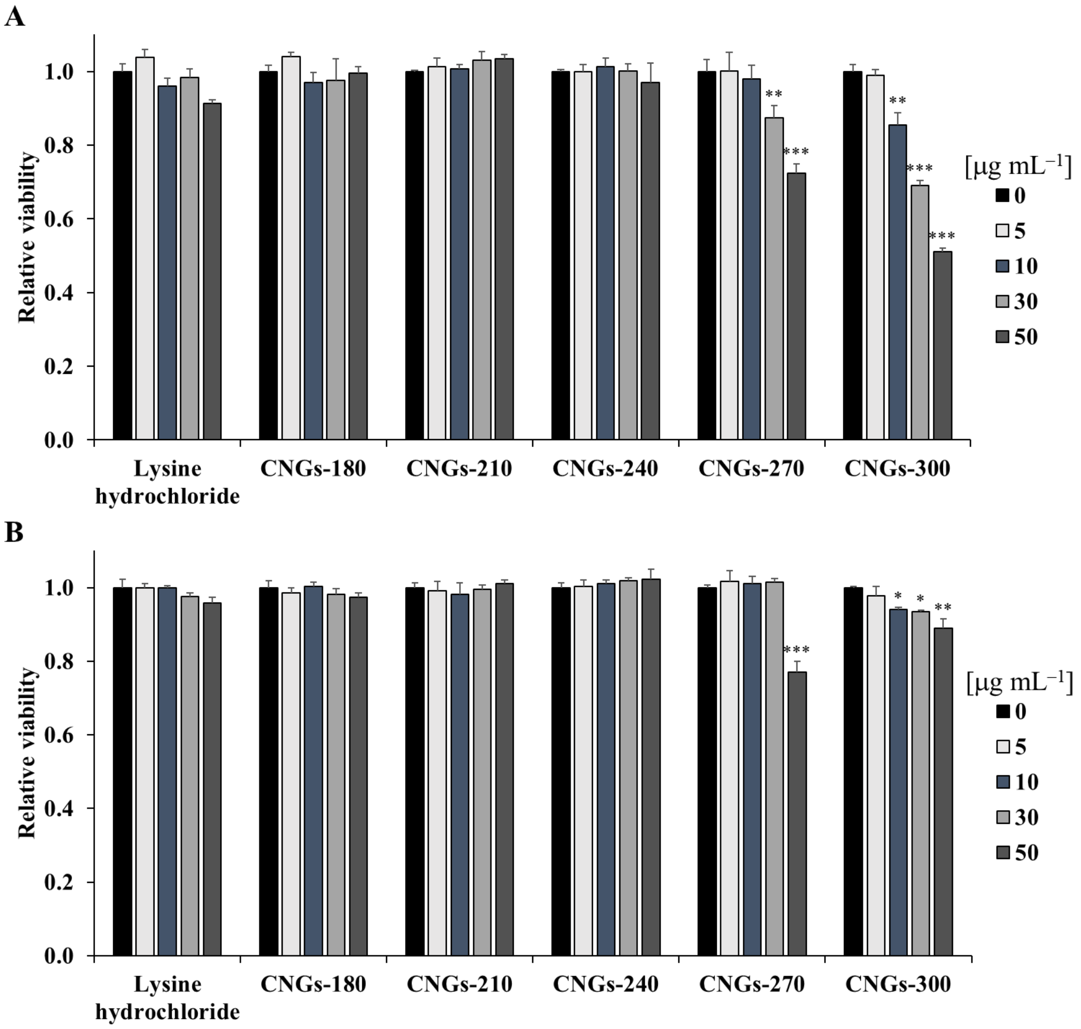

Figures S5. Relative cell viability of (A) BHK-21 cells and (B) Vero cells treated with lysine hydrochloride or CNGs with the concentration of 5 to 50 μg mL^{-1} in media for 30 min followed by replacing the CNGs with fresh media and cultured for 72 h. Error bars represent the standard deviation of four separate experiments. (*$p < 0.05$, **$p < 0.01$, ***$p < 0.001$ versus cell control).

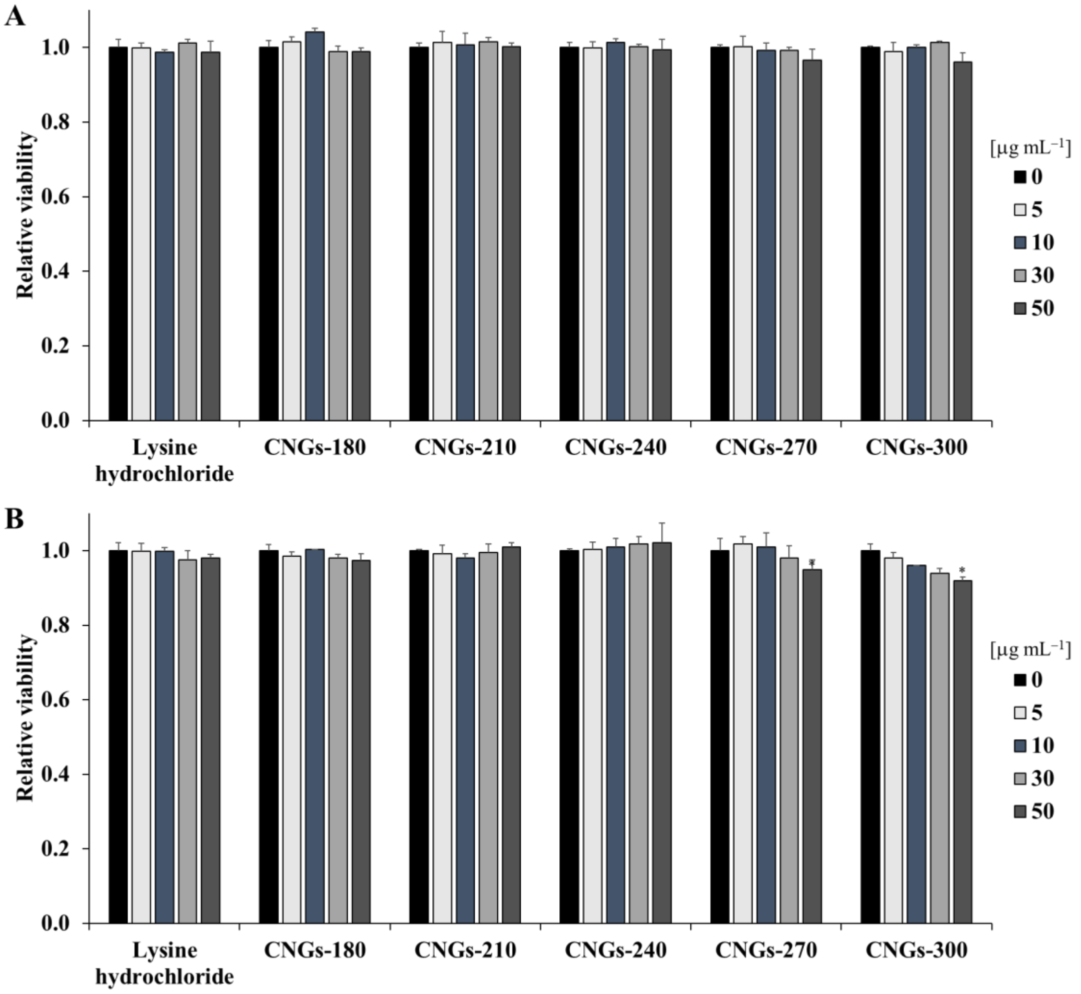

Figure S6. Relative cell viability of (A) BHK-21 cells and (B) Vero cells treated with lysine hydrochloride or CNGs with the concentration of 5–50 μg mL^{-1} in media for 24 h. Error bars represent the standard deviation of four separate experiments. (*$p < 0.05$, versus cell control).

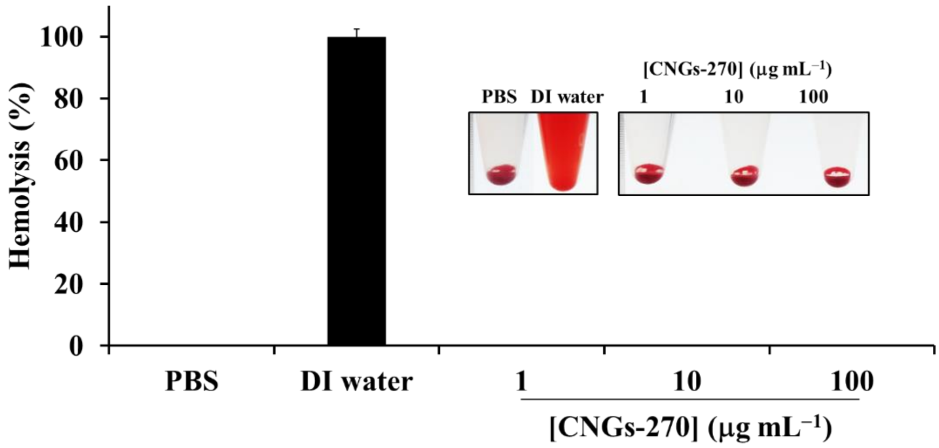

Figure S7. Hemolytic activities of CNGs-270 (1–100 μg mL^{-1}) on RBCs. Hemolysis assay with PBS and DI water was used as a negative control and positive control, respectively. Error bars represent the standard deviation from three separate experiments.

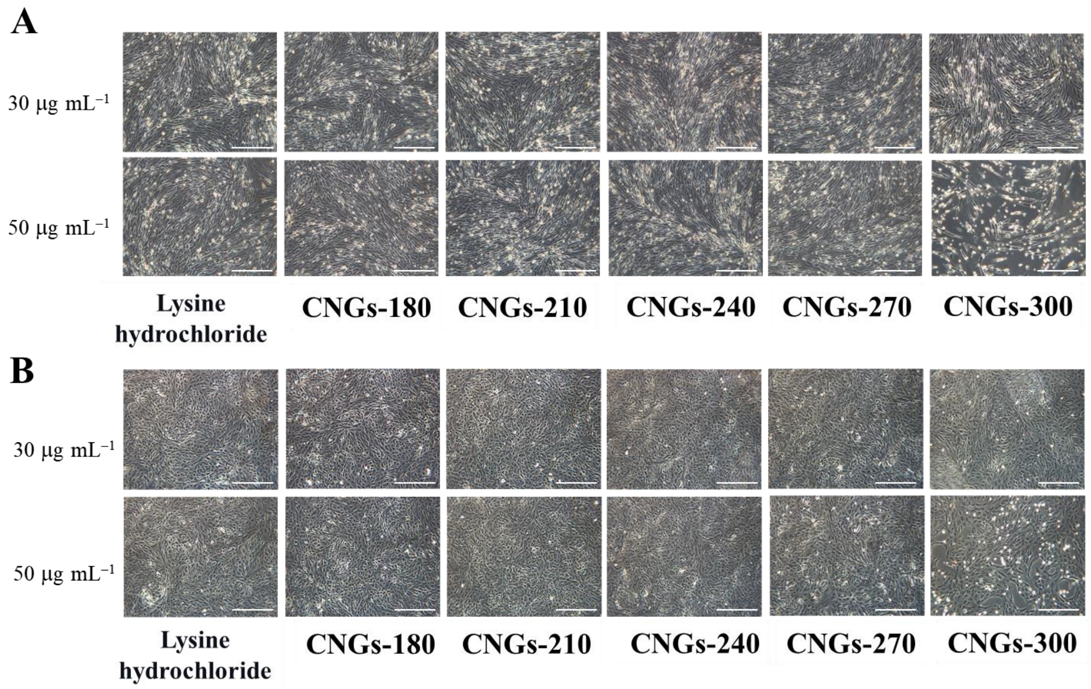

Figure S8. Microscopic images of (A) BHK-21 cells and (B) Vero cells treated with lysine hydrochloride or CNGs with a concentration of 30 or 50 μg mL^{-1}, followed by replacing fresh medium and cultured for 72 h. The scale bar in the images is 500 μm.

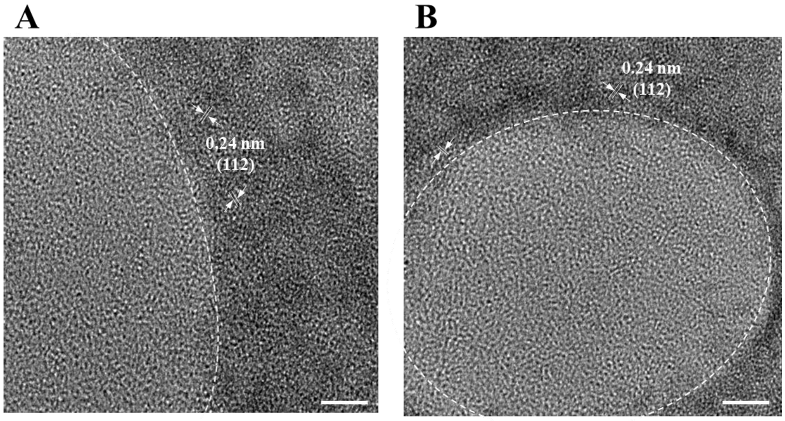

Figure S9. HRTEM images of (A) BEFV ($5 \times 10^{5.5}$ $TCID_{50}$ mL^{-1}) and (B) PRV ($5 \times 10^{5.5}$ $TCID_{50}$ mL^{-1}) after incubation with CNGs-270 (1.0 μg mL^{-1}) for 30 min in PBS. The circle region is the virus particle. The scale bar in the HRTEM images is 5 nm.
